# Efficacy of Acupuncture in Children with Nocturnal Enuresis: A Systematic Review and Meta-Analysis of Randomized Controlled Trials

**DOI:** 10.1155/2015/320701

**Published:** 2015-06-16

**Authors:** Zheng-tao Lv, Wen Song, Jing Wu, Jun Yang, Tao Wang, Cai-hua Wu, Fang Gao, Xiao-cui Yuan, Ji-hong Liu, Man Li

**Affiliations:** ^1^Department of Orthopedics, Tongji Hospital, Tongji Medical College, Huazhong University of Science and Technology, Wuhan 430030, China; ^2^Department of Urology, Tongji Hospital, Tongji Medical College, Huazhong University of Science and Technology, Wuhan 430030, China; ^3^Key Laboratory of Environment and Health, Ministry of Education and Department of Epidemiology and Biostatistics, School of Public Health, Tongji Medical College, Huazhong University of Science and Technology, Wuhan 430030, China; ^4^Department of Neurobiology, School of Basic Medicine, Tongji Medical College of Huazhong University of Science and Technology, Wuhan 430030, China

## Abstract

*Background*. Nocturnal enuresis (NE) is recognized as a widespread health problem in young children and adolescents. Clinical researches about acupuncture therapy for nocturnal enuresis are increasing, while systematic reviews assessing the efficacy of acupuncture therapy are still lacking. *Objective*. This study aims to assess the effectiveness of acupuncture therapy for nocturnal enuresis. *Materials and Methods*. A comprehensive literature search of 8 databases was performed up to June 2014; randomized controlled trials which compared acupuncture therapy and placebo treatment or pharmacological therapy were identified. A meta-analysis was conducted. *Results*. This review included 21 RCTs and a total of 1590 subjects. The overall methodological qualities were low. The results of meta-analysis showed that acupuncture therapy was more effective for clinical efficacy when compared with placebo or pharmacological treatment. Adverse events associated with acupuncture therapy were not documented. *Conclusion*. Based on the findings of this study, we cautiously suggest that acupuncture therapy could improve the clinical efficacy. However, the beneficial effect of acupuncture might be overstated due to low methodological qualities. Rigorous high quality RCTs are urgently needed.

## 1. Introduction

Nocturnal enuresis (NE) is a worldwide health problem frequently encountered in childhood and is defined as an involuntary voiding of urine during sleep with a frequency of at least twice a week in children, in the absence of congenital or acquired defects of the central nervous system [1]. It includes monosymptomatic nocturnal enuresis (MNE) with no daytime urinary symptoms and nonmonosymptomatic nocturnal enuresis (NMNE) that is accompanied by daytime urinary symptoms. Nocturnal enuresis affects 5%–10% of younger school-age children [2]. Enuretic children have a higher risk for psychosocial comorbidity and loss of self-esteem. Such feelings of humiliation, guilt, and shame are also a reasonable source of heartache to the children and their parents. The etiology and underlying physiological mechanisms of nocturnal enuresis are multifactorial; three commonly proposed mechanisms to bedwetting include excessive nocturnal urine production, bladder overactivity, and a failure to awaken in response to bladder sensations [3].

Current first-line nocturnal enuresis therapies center on the aforementioned mechanisms; generally accepted treatments are oral pharmacological therapies including desmopressin, tricyclics, or oxybutynin and behavioral therapies [4]. Desmopressin has been widely used for several decades, and its reliable therapeutic effect has been proven to one-third of the unselected enuretic children. But the clinical effect cannot be maintained once the medication is stopped and the side effects associated with drugs may cause the patients to be reluctant to use them for long periods. The preferred behavioral treatment is bed alarm, which needs to be continuous and brings the enuretic children different degrees of sleep disorders at the same time [5].

Complementary and alternative medicine (CAM) is widely advocated to face the increasing demand for nonpharmacological approaches. As a mainstream CAM therapy, acupuncture treatment based on TCM theory has been commonly used to treat nocturnal enuresis in Chinese cultures. Compared to conventional care, its safety and cost effectiveness assure the maintenance of patients' compliance. Unfortunately, there is little published information to warrant acupuncture therapy as standard treatment of nocturnal enuresis. The aim of this review is to evaluate the efficacy of acupuncture therapy in the treatment of nocturnal enuresis when compared with placebo acupuncture or oral pharmacological treatment based on randomized controlled trials (RCTs).

## 2. Material and Methods

### 2.1. Literature Search Strategy

A comprehensive literature search of the Cochrane Central Register of Controlled Trials (CENTRAL), Cochrane Database of Systematic Review (CDSR), EMBASE, ISI Web of Science, and PubMed was conducted. We also searched Chinese databases, including China Knowledge Resource Integrated database (CNKI), WanFang Data, VIP, and Chinese Biomedical (CBM) Literature database. In addition, we searched databases that contained registered trials, such as ClinicalTrials.gov (http://www.clinicaltrials.gov). All databases were searched from their inception dates up to June 2014; languages were restricted to Chinese and English. The following medical subject headings or key words were used for English databases: enuresis, nocturnal enuresis, nighttime urinary incontinence, bedwetting, acupuncture, electroacupuncture, auricular acupuncture, ear acupuncture, scalp acupuncture, acupoint, moxibustion, acupressure, and acustimulation. For Chinese databases we used free text terms as “Zhen” or “Jiu” or “Xue Wei” and “Yi Niao.” In addition, the bibliographies of relevant systematic reviews and clinical guidelines were manually searched. We also searched the gray literature that included dissertations, letters, government documents, research reports, conference proceedings, and abstracts when available. The reference section for each study was also searched.

### 2.2. Inclusion Criteria

Inclusion criteria are as follows: (1) research subjects: the enrolled patients had to be diagnosed with NE and no restrictions on race, age, or sex were imposed; (2) study design: the included studies were required to be RCTs in Chinese or English aiming to assess the efficacy of acupuncture therapy for NE; (3) experimental group interventions: experimental group mainly received acupuncture therapy (including needles, moxibustion, acupressure, electroacupuncture, and acupoint injection, among other techniques), either alone or in conjunction with another kind of acupuncture therapy, without differentiating different acupuncture therapy techniques, acupoints selection, or needle materials; (4) control group interventions: control interventions included placebo acupuncture or oral pharmacological treatment; (5) outcome measurements: the outcome measurement had to include overall clinical efficacy, number of wet nights per week, or maximum voided volume.

### 2.3. Exclusion Criteria

Exclusion criteria included the following: (1) articles regarding animal experiments, review articles, case reports, or expert experience reports; (2) nonrandomized studies; (3) studies that compared different acupuncture modalities or acupoints selection; (4) experimental groups that accepted complex therapy, while the contributing factor could not be distinguished; (5) studies that were duplicates for retrieving or publishing.

### 2.4. Data Extraction

Two reviewers (Zheng-tao Lv and Wen Song) reviewed each article independently and were blinded to the findings of the other reviewer. In accordance with the predetermined inclusion criteria, two reviewers independently performed a rigorous screening to identify qualified articles, and they extracted data independently from these articles using a standardized collection form, which includes first author, year of the study, sample size, nation or region, baseline characteristics, methodological features of the studies, quality of trial design, interventions, main outcome assessments, follow-up time, and withdrawal. If the required information was not available in the included studies, we contacted the original authors by email. Any discrepancies between reviewers were resolved through discussion until a consensus was reached. The third review author (Man Li) was consulted if a consensus could not be reached.

### 2.5. Quality of the Studies

The methodological quality of the included trials was evaluated using the Jadad quality scale [27]: (1) randomization (the study was described as randomized), (2) double blinding (participant masking and researcher masking), (3) reporting of the number of dropouts and reasons for withdrawal, (4) allocation concealment, and (5) generation of random numbers (by using computer, random numbers table, shuffled cards, or tossed coins). RCTs scored 1 point for each area addressed in the study design for a possible score between 0 and 5 (highest level of quality). The quality of all included studies was assessed by two authors (Zheng-tao Lv and Wen Song) and the articles were classified as high quality if their Jadad score ≥4 and low quality if their Jadad score ≤3. Disagreements regarding methodological quality were resolved with discussion between reviewers.

### 2.6. Data Synthesis and Analysis

The meta-analysis and statistical analysis were performed by using RevMan 5.1 analyses software of the Cochrane Collaboration. We extrapolated the odds ratio (OR) and the associated 95% confidence interval (CI) for treatment effect. The chi-squared test and the Higgins *I* ^2^ test were used to assess the heterogeneity of the data [28]. We pooled data across studies using random effect models if statistical heterogeneity exists; otherwise, a fixed effect model will be used. Publication bias was explored via a funnel-plot analysis. Begg's test and Egger's test were conducted when the number of included studies is equal to or greater than 5 (Stata Software, version 12.0). In case of heterogeneity, subgroup analysis was conducted.

## 3. Results

### 3.1. Literature Search Results

**Figure 1:**
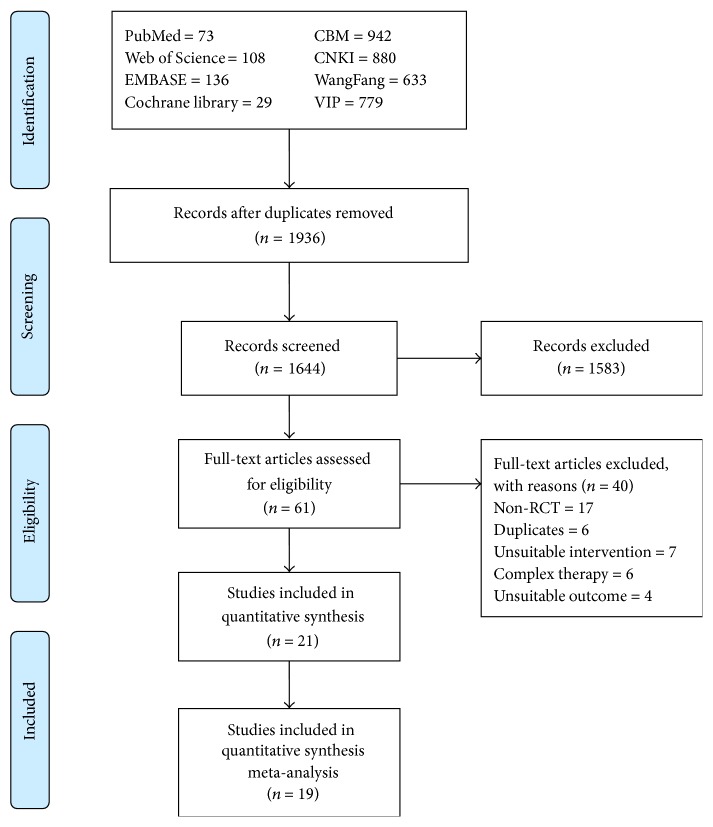
Flowchart of the literature search and study selection.

An initial search of RCTs yielded 3580 potential literature citations, including 346 English studies and 3134 Chinese studies, and 1936 duplicated articles were deleted. After screening titles and abstracts, 61 potentially relevant studies were selected and retrieved for a full-text assessment. Of the remaining 61 studies, 17 studies were excluded because they were not RCTs; 6 articles were duplicates; 7 studies took unsuitable intervention; 6 studies accept the complex therapy, for example, combination of two different kinds of acupuncture therapy; 4 studies did not report the suitable outcome. Finally, 21 studies meet our inclusion criteria [6–26]. Because only two of these studies [10, 17] compared acupuncture with placebo treatment (e.g., without active laser light but with or without skin contact), we just used them for systematic review (Figure 1).

### 3.2. The Characteristics and Methodological Quality of the Included Trials

The characteristics of the 21 trials are summarized in Table 1. These studies were published between 2001 and 2014. Sixteen studies were published in Chinese and five studies in English. The 21 RCTs included a total of 1590 patients with nocturnal enuresis: 826 patients in the acupuncture group (experimental group) and 731 patients in the control group. Age of the patients ranges from 3 to 21 years. Nineteen studies used 2-parallel-arm group designs [6, 8–12, 14–26] and two used a 3-parallel-arm group design [7, 13]. The experimental group mainly received acupuncture therapy (including needles, moxibustion, acupressure, electroacupuncture, and acupoint injection, among other techniques). Among the 21 studies, western medicine therapy (e.g., desmopressin, Meclofenoxate) was used as the intervention method for the control group in 9 studies [6, 8, 9, 11, 13, 16, 22, 23, 25]; traditional Chinese medicine (TCD) was used in 10 studies [7, 12, 14, 15, 18–21, 24, 26]; and placebo treatment or sham-acupuncture was used in 2 studies [10, 17]. The main outcome indicators reported in the included studies were cure rate, improvement rate, and mean weekly number of wet nights; Two studies reported maximum voided volume (MVV) as outcome indicators [13, 17] (Table 2).

The mean Jadad score of these 21 studies was 1.7, ranging from 1 to 4 points (Table 1). Only 1 of 21 RCTs met the Jadad criteria for high quality [17]. All of the studies included suggested randomization, and 9 studies reported the method of random sequences generation [7, 8, 12–17, 20]. In that study, it was not feasible to blind the participant or the therapist. The outcome assessor was blinded in only two studies [10, 17]; we considered that the outcomes and their measurements are likely to be influenced by lack of blinding. Four studies reported complete follow-up of all subjects [10, 13, 14, 17]. All the studies presented selective reporting, characterized similarity of baseline. In general, the methodological and report qualities of the included studies were poor.

### 3.3. Meta-Analysis Results

The 21 included RCTs adopted in consistent interventions and different reported outcomes, with no unified efficacy standard. To reach a consistent understanding of the therapeutic effect of acupuncture therapy for nocturnal enuresis, intervention therapies for control group were further refined. We limited the control group methods to western or traditional Chinese medicine alone, as two studies used placebo treatment or sham-acupuncture as control group [10, 17] and one of these two studies did not report the cure rate as effective outcomes [17]. Furthermore, the definition of cure rate was consistent among the other included 19 studies; we conducted the meta-analysis to compare the overall cure rate determined in these studies.

Three studies reported mean weekly number of wet nights [10, 13, 17] and two studies reported maximum voided volume (MVV) [13, 17] as the effective outcomes, considering the lack of adequate numbers of studies; these results will be presented in the following part of our review.

**Figure 2:**
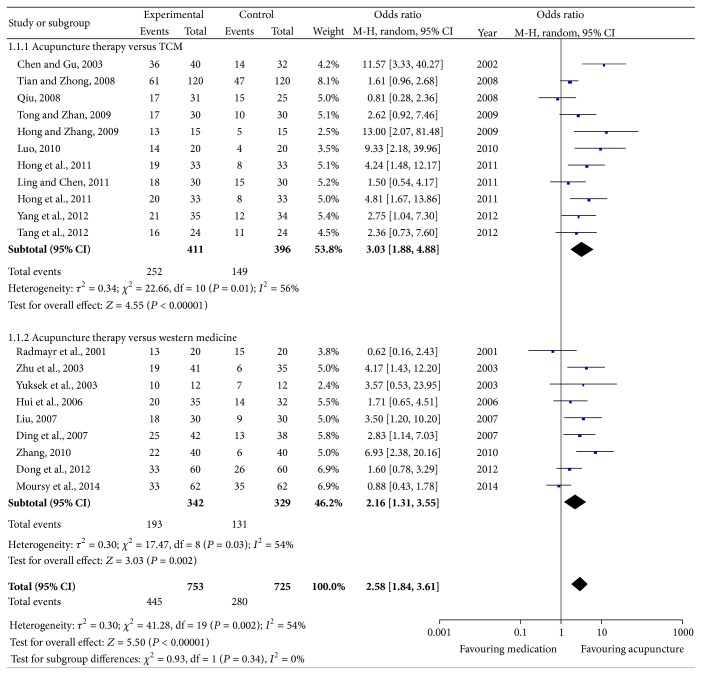
Forest plot of comparison: the clinical effective rate.

The results of heterogeneity tests indicated that *I* ^2^ > 50% and *P* < 0.1 for the 19 included studies [6–9, 11–16, 18–26] and that the overall heterogeneity existed (*P* = 0.002, *I* ^2^ = 54%). Therefore, a random effects model was used. The combined effects of 19 independent trial results showed that acupuncture therapy had further improved the cure rate in patients with nocturnal enuresis when compared with control group accepting medicine therapy (OR = 2.58; 95% CI, 1.84–3.61; *P* < 0.0001) (Figure 2).

#### 3.3.1. Acupuncture versus Western Medicine

Our meta-analysis of ten studies [6, 8, 9, 11, 13, 16, 22, 23, 25], which compared acupuncture therapy with traditional Chinese medicine, yielded encouraging effects in favor of acupuncture therapy on nocturnal enuresis (OR = 2.16; 95% CI, 1.31–3.55; *P* < 0.01). Heterogeneity between studies existed (*P* = 0.03; *I* ^2^ = 54%) (Figure 2).

#### 3.3.2. Acupuncture versus Traditional Chinese Medicine

The same findings applied to other ten studies [7, 12, 14, 15, 18–21, 24, 26], which compared acupuncture therapy with western medicine, yielded encouraging effects in favor of acupuncture therapy on nocturnal enuresis (OR = 3.03; 95% CI, 1.88–4.88; *P* < 0.01). Heterogeneity between studies existed (*P* = 0.01; *I* ^2^ = 56%) (Figure 2).

### 3.4. Subgroup Analyses

**Figure 3:**
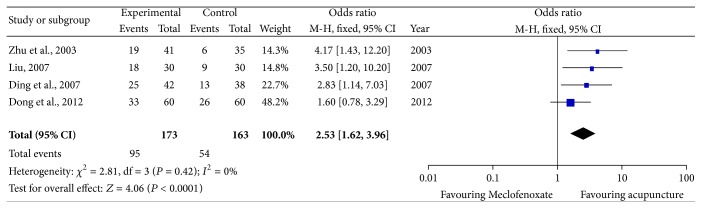
Subgroup analysis: acupuncture therapy versus Meclofenoxate.

**Figure 4:**
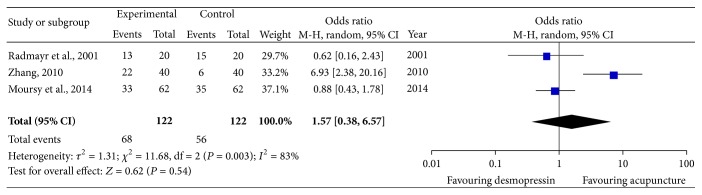
Subgroup analysis: acupuncture therapy versus desmopressin.

A subgroup analysis was conducted to further evaluate the clinical effect of acupuncture therapy and identify the heterogeneity within western medicine group. The western medicine group was divided into four groups according to the medication types. Four studies used Meclofenoxate as control intervention [6, 9, 11, 22], three studies used desmopressin as medicine control [13, 16, 23], and the remaining two studies [8, 25] treated nocturnal children with imipramine hydrochloride and oxybutynin, respectively. The pooled data showed significant difference between acupuncture therapy and Meclofenoxate (OR = 2.81; 95% CI, 1.62–3.96; *P* < 0.0001), with no obvious heterogeneity (Figure 3). The pooled effects of three independent trials suggested that there was no significant difference between desmopressin and acupuncture in treating NE (OR = 1.57; 95% CI, 0.38–6.57; *P* = 0.54) (Figure 4). Since only one trial utilized imipramine hydrochloride as medicine control and only one trial utilized oxybutynin, results from these two studies are presented as narrative description. There was no significant difference between imipramine hydrochloride and acupuncture therapy (OR = 1.71; 95% CI, 0.65–4.51; *P* = 0.27). Compared with oxybutynin, acupuncture could not further improve the clinical effect (OR = 3.57; 95% CI, 0.53–2; *P* = 0.54).

### 3.5. Acupuncture Therapy versus Placebo Treatment

Two studies used placebo treatment or sham-acupuncture as control group [10, 17]. However, results of these two studies were inconsistent. Radvanska et al. [17] compared the treatment efficacy of laser acupuncture therapy with sham-acupuncture; they found no significant effect of active laser acupuncture on maximal voided volume (first morning void excluded), maximal morning voided volume, voiding frequency, enuresis frequency before and after treatment, or nocturnal urine production among the patient groups, but it resulted in a significant increase in average daytime voided volume. There was no effect of skin contact during placebo laser acupuncture. Radvanska et al. [17] concluded that laser acupuncture had subtle effects on bladder reservoir function; however, it is an inefficient treatment for monosymptomatic nocturnal enuresis with reduced maximal voided volume. Karaman et al. [10] evaluated the effect of laser acupuncture therapy on patients with primary monosymptomatic nocturnal enuresis. The mean number of bedwetting episodes was 1.7 per week 6 months after laser therapy and 3.1 in the placebo group. Laser acupuncture therapy was significantly more beneficial compared to placebo in terms of complete dryness, partial improvement, and decrease in the mean number of weekly bedwetting episodes.

### 3.6. Other Outcomes

#### 3.6.1. Mean Weekly Number of Wet Nights

Three studies reported mean weekly number of wet nights [10, 13, 17]. Moursy et al. [13] reported that the difference of reducing the mean weekly number of wet nights in laser acupuncture group, desmopressin group, and combination of laser acupuncture and desmopressin group had no statistical significance (*P* > 0.05). Radvanska et al. [17] found that the difference in the reduction of wet nights was not statistically significant between laser acupuncture group and placebo group. Karaman et al. [10] showed that laser acupuncture therapy was significantly more beneficial compared to placebo in terms of a decrease in the mean number of weekly bedwetting episodes as previously mentioned.

#### 3.6.2. Maximum Voided Volume (MVV)

Two studies reported maximum voided volume (MVV) [13, 17] as the effective outcomes. Moursy et al. [13] found that it significantly increased only in laser acupuncture group and combination of laser acupuncture and desmopressin group comparing with pretreatment values and desmopressin group, respectively. Thus, bladder capacity significantly increased only in patients receiving laser acupuncture treatment. However, Radvanska et al. [17] reported that the MVV had no difference between laser acupuncture group and placebo group.

### 3.7. Publication Bias Analysis

**Figure 5:**
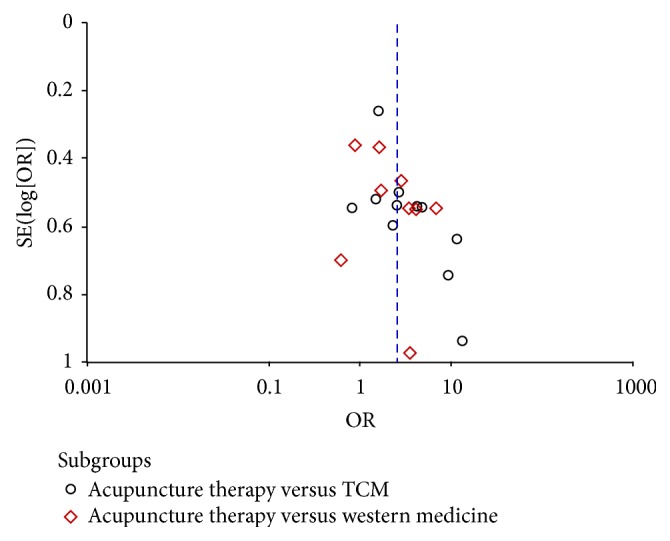
Funnel plot of randomized controlled trials.

We conducted a funnel plot analysis of the aforementioned 19 studies [6–9, 11–16, 18–26]. *P* value associated with Begg's test was 0.009 and *P* value associated with Egger's test was 0.002. The resulting graph was asymmetrical, suggesting the possibility of publication bias, which was in line with results of Begg's test and Egger's test (Figure 5). In addition, language bias may exist because most of included studies were published in Chinese.

## 4. Discussion

### 4.1. Summary of Evidence

The present study analyzed data from 21 RCTs involving 1590 individuals that featured to assess the efficacy of acupuncture therapy to treat NE. Based on the findings in our systematic review and meta-analysis, acupuncture therapy can significantly improve the clinical efficacy in enuretic children when compared with placebo acupuncture or TCM. In contrast to western medicine, acupuncture therapy was more effective than Meclofenoxate. Conclusions regarding the safety of acupuncture therapy cannot be drawn due to the paucity of evidence provided by the included trials. However, the drawn conclusion should be interpreted cautiously owing to low methodological qualities of included studies.

### 4.2. Mechanism of Acupuncture Therapy

The pathogenesis of nocturnal enuresis is multifactorial; several factors such as psychosocial, developmental, hormonal, and genetic factors have been proven to be involved in nocturnal enuresis. Nocturnal polyuria, nocturnal detrusor overactivity, and high arousal thresholds are main pathogenesis of NE. To date, increasing evidence suggests that all three mechanisms can be attributed to brainstem disturbance. The locus coeruleus (LC) has axonal connections with the hypothalamic cells that produce vasopressin and also plays an important role in arousal from sleep [29, 30]. Pontine micturition center coordinates the micturition reflex and overlaps both functionally and anatomically with LC. A disturbance in this region of brainstem may cause a range of pathological changes which may result in the pathogenesis of NE.

Acupuncture points were selected in order to influence the spinal micturition centers as well as the parasympathetic innervation to the urinary tract [31]. With acupuncture stimulation, levels of enkephalins and endogenous opioids are increased in both plasma and central nervous system. An increased beta-endorphin level in human cerebrospinal fluid could be detected after acupuncture stimulation [32]. And beta-endorphin was found to be able to depress bladder contractions [33]. The therapeutic effects of acupuncture therapy can be achieved through the suppression of spinal and supraspinal reflexes which lead to bladder contraction. And the clinical efficacy of acupuncture was reflected in increase in maximum bladder capacity and suppression of detrusor muscle activity; these functional changes might contribute to the improvement of NE.

In TCM theory, the generation and discharge of urine are associated with lung, kidney, spleen, and bladder. The pathogenesis of nocturnal enuresis is Qi deficiency of lung, spleen, and kidney; bladder is not controlled by Qi as well. Through different forms of stimulation on meridian points or specific parts of body, imbalance and instability between Zangfu organs are corrected to improve symptoms of NE and maintain the stability of inner state [34]. Based on the classical prescriptions of acupuncture, series of novel acupuncture modalities have been widely applied in clinic. In our systematic review, the specific interventions employed in these eligible trials included traditional fine needle acupuncture, moxibustion, electroacupuncture, auricular point sticking, acupoint catgut embedding, acupressure, transdermal drugs delivery systems, and acupoint injection. These techniques were considered as one type of therapy, without differentiating acupoint selection or acupuncture forms. Therefore, the findings in this review might indicate an overall efficacy trend, but definitive conclusions could not be drawn.

### 4.3. Comparison with Other Studies

In 2005, a systematic review reported that acupuncture in combination with another therapy could further significantly reduce the number of wet nights when compared to acupuncture therapy alone, and, regarding the comparison of acupuncture therapy with antidiuretic medication, the results showed that the outcome favored medication but was not significantly better than acupuncture therapy [35]. Our meta-analysis managed to summarize all published RCTs to compare the clinical efficacy of acupuncture therapy with pharmacological treatment or placebo treatment. The findings in our meta-analysis suggested that acupuncture therapy was more effective than both western diuretic medication and traditional Chinese medicine, which ran counter to the conclusion in aforementioned systematic review.

### 4.4. Limitations

Based on the studies included in our meta-analysis, the methodological qualities were judged to be generally poor, which might limit the value of conclusions about clinical efficacy of acupuncture therapy for treating NE. The vast majority of the included trials failed to describe detailed information about randomization and allocation concealment. Lack of blinding procedures in RCTs can also exaggerate the conclusions of these trials. Further assessment of acupuncture therapy needs to be taken by large-scale clinical studies which employ rigorous methodologies.

The diagnosis and therapeutic evaluation standards employed by studies, that are performed in China, are mainly in accordance with “Standards for Diagnosis of Syndromes or Diseases of TCM and Evaluation of the Therapeutic Effect” issued by the State Administration of TCM in 1994 [36]. In the studies published in English, the majority of recruited patients are diagnosed and evaluated according to the “Standardization and Definition of Lower Urinary Tract Dysfunction in Children” of the International Children's Continence Society (ICCS) [37]. To conduct a meta-analysis, the outcome measure adopted in included RCTs was clinical efficacy. Such terms, cure rate, complete improvement rate, and response rate, are synonyms; children having no bedwetting episodes on follow-ups were defined to be cured. The majority of our eligible studies failed to distinguish between NMNE and MNE, making it difficult to get a precise conclusion. To our knowledge, there is still no worldwide unified evaluation standard to assess the basic state and disease's progression of enuretic children. In addition, the duration of acupuncture sessions and follow-ups after treatments vary from studies to studies. Since acupuncture therapy has a long-lasting beneficial effect on enuretic children, the outcomes were supposed to be measured at the end of follow-ups after treatment.

The utilization of different acupuncture techniques by different investigator can greatly affect curative effect of acupuncture therapy [5]. Based on TCM theory, all acupuncture procedures need to be performed according to syndrome differentiation. A lack of understanding of TCM was reflected in the treatment models; treatment following the same pattern can reduce the therapeutic effect to some extent. Acupuncture sessions should be performed based upon strict diagnosis made by four basic diagnostic methods (inspection, auscultation, olfaction, and palpation) [38]. As various acupuncture modalities are difficult to master, practitioners and physicians are required to have a deep understanding of the mechanisms underlying NE so that acupuncture techniques could be applied appropriately. The investigators who lack universal knowledge of TCM theory should be encouraged to participate in the standardized training before the application of acupuncture.

In contrast to TCM, acupuncture therapy could further improve the clinical effect in treating nocturnal children; no subgroup analysis was made in this group because the acupuncture modalities and Chinese medicine types varied from studies to studies. The data extracted from these studies suggested an overall efficacy trend; the standardization of acupuncture techniques is one problem to be solved in need. In the subgroup analysis conducted in western medicine group, acupuncture therapy was more effective than Meclofenoxate while no significant difference could be detected between acupuncture and imipramine hydrochloride, desmopressin, or oxybutynin. Types and doses of administered drugs might affect the results of experiment to a certain extent. Given that the evidence from China occupies a large proportion, further rigorous experiments within western context are required. Considering all these above factors, the appearance of heterogeneity could be reasonably explained.

### 4.5. Suggestion for Future Research

The included studies in our systematic review comprise various methodological deficiencies, and the findings of the present review are somewhat limited due to low methodological qualities. Future randomized controlled trials should employ improved methodologies and reporting specifications as follows: (1) all clinical studies of acupuncture should be registered and comply with the revised standards for reporting interventions in clinical trials of acupuncture (STRICTA) [39]; (2) the sample sizes should be calculated; (3) the generation of random allocation sequences and allocation concealment should be provided in detail; (4) these studies should be blinded and placebo controlled; (5) the standard of diagnosis should be unified and widely accepted; (6) the follow-ups after treatments are required to be at least 6 months so that patients could be revalued; (7) all adverse events associated with acupuncture should be reported and rigorously assessed.

## 5. Conclusion

In summary, the results of this study suggest that acupuncture therapy demonstrate better clinical efficacy than pharmacological treatment or placebo treatment in treating NE. Due to the low methodological qualities of included trials, the findings of current study should be interpreted with caution. Therefore, to further assess the potential beneficial effect of acupuncture therapy for NE, additional RCTs with rigorous experimental design, large-scale high quality methodological control, long follow-ups, and strict reporting specification are required.

## Figures and Tables

**Table 1:** Characteristics and methodological quality of included studies.

Study	Study design	Sample size (*n*1/*n*2)	Nation/region	Age (mean or range)	Baseline	EC approval	Jadad score
Dong et al., 2012 [6]	RCT, parallel 2 arms	120 (60/60)	China	E: 8.61 (5~12) years C: 8.57 (5~13) years	Adequate	Not reported	1
Hong et al., 2011 [7]	RCT, parallel 3 arms	99 (33/33/33)	China	5~13 years	Adequate	Not reported	2
Hui et al., 2006 [8]	RCT, parallel 2 arms	67 (35/32)	China	E: 5~12 years C: 6~11 years	Adequate	Not reported	2
Liu, 2007 [9]	RCT, parallel 2 arms	60 (30/30)	China	5~12 years	Not reported	Not reported	1
Karaman et al., 2011 [10]	RCT, parallel 2 arms Prospective, randomized, placebo controlled, single-blind study	83 (57/26)	Turkey	E: 8.5 ± 3.2 years C: 8.9 ± 3.3 years	Adequate	Yes	3
Ding et al., 2007 [11]	RCT, parallel 2 arms	80 (42/38)	China	3~13 years	Adequate	Not reported	1
Tong and Zhan, 2009 [12]	RCT, parallel 2 arms	60 (30/30)	Guinea-Bissau	6~20 years	Adequate	Not reported	2
Moursy et al., 2014 [13]	RCT, parallel 3 arms	186 (62/62/62)	Egypt	15.7 years (range 10–21 years)	Adequate	Yes	3
Tian and Zhong, 2008 [14]	RCT, parallel 2 arms	228 (116/112)	China	E: 7.58 ± 2.16 years C: 8.26 ± 2.67 years	Adequate	Not reported	3
Ling and Chen, 2011 [15]	RCT, parallel 2 arms	60 (30/30)	China	E: 9.2 (5~16) years C: 9.1 (5~15) years	Adequate	Not reported	2
Radmayr et al., 2001 [16]	RCT, parallel 2 arms	40 (20/20)	Austria	E: 8.6 (5~16) years C: 8.0 (5~14) years	Adequate	Yes	2
Radvanska et al., 2011 [17]	RCT, parallel 2 arms Prospective, single-blind, randomized, placebo controlled design	29 (16/13)	Slovakia	E: 8.7 ± 1.4 years C: 8.6 ± 1.3 years	Adequate	Yes	4
Yang et al., 2012 [18]	RCT, parallel 2 arms	69 (35/34)	China	3~15 years	Adequate	Not reported	1
Luo, 2010 [19]	RCT, parallel 2 arms	40 (20/20)	China	E: 8.5 ± 0.1 years C: 8.4 ± 0.2 years	Adequate	Not reported	1
Tang et al., 2012 [20]	RCT, parallel 2 arms	48 (24/24)	China	E: 5~11 years C: 5~12 years	Adequate	Not reported	2
Qiu, 2008 [21]	RCT, parallel 2 arms	56 (31/25)	China	3~16 years	Not reported	Not reported	1
Zhu et al., 2003 [22]	RCT, parallel 2 arms	76 (41/35)	China	4~15 years	Adequate	Not reported	1
Zhang, 2010 [23]	RCT, parallel 2 arms	80 (40/40)	China	3~18 years	Adequate	Not reported	1
Chen and Gu, 2003 [24]	RCT, parallel 2 arms	72 (40/32)	China	5~14 years	Adequate	Not reported	1
Yuksek et al., 2003 [25]	RCT, parallel 2 arms	24 (12/12)	Turkey	E: 7.67 ± 2.34 years C: 7.41 ± 2.67 years	Adequate	No	1
Hong and Zhang, 2009 [26]	RCT, parallel 2 arms	30 (15/15)	China	8~21 years	Adequate	Not reported	1

**Table 2:** Interventions and outcomes of included studies.

Study	Duration of treatment	Follow-up after treatment	Experimental treatment	Control treatment	Cure rate of intervention group	Cure rate of control group	Outcome measurement
Dong et al., 2012 [6]	5 weeks	6 months	Acupoint injection with scraping therapy (*n* = 60)	Western medicine: Meclofenoxate (*n* = 60)	46/60 (76.67%)	36/60 (60%)	Cure rate, improvement rate, follow-up at 1 and 6 months

Hong et al., 2011 [7]	1 month	Not reported	Moxibustion (*n* = 33) Acupuncture (*n* = 33)	Chinese patent medicine (*n* = 33)	20/33 (60.6%) 19/33 (57.6%)	8/33 (24.24%)	Cure rate, improvement rate

Hui et al., 2006 [8]	1 month	1 year	Heat-producing needling (*n* = 35)	Western medicine: imipramine hydrochloride (*n* = 32)	20/35 (57.2%)	14/32 (43.8%)	Cure rate, total effective rate, follow-up at 1 month

Liu, 2007 [9]	3 weeks	Not reported	Enuresis patch (*n* = 30)	Western medicine: Meclofenoxate (*n* = 30)	18/30 (60%)	9/30 (30%)	Cure rate, improvement rate

Karaman et al., 2011 [10]	4 weeks	6 months	Laser acupuncture (*n* = 57)	Placebo therapy: with a nonlaser light source (*n* = 26)	31/57 (54.4%)	3/26 (11.5%)	Complete improvement rate, partial improvement rate, mean number of weekly bedwetting episodes: the children were reevaluated 15, 30, 90, and 180 days after treatment

Ding et al., 2007 [11]	1 month	3 months	Enuresis patch (*n* = 42)	Western medicine: Meclofenoxate (*n* = 38)	25/42 (59.5%)	13/38 (34.2%)	Cure rate, improvement rate

Tong and Zhan, 2009 [12]	1 month	Not reported	Suspended moxibustion (*n* = 30)	Chinese patent medicine (*n* = 30)	17/30 (56.7%)	10/30 (33.3%)	Cure rate, improvement rate

Moursy et al., 2014 [13]	3 months	6 months	Laser acupuncture (*n* = 62)	Western medicine: desmopressin (*n* = 62) Combination therapy: acupuncture + desmopressin (*n* = 62)	33 /62 (53%)	35/62 (56.5%) 46/82 (74%)	Cure rate, improvement rate, mean weekly number of wet nights, MVV (maximum voided volume): the patients were evaluated once every 2 weeks for 3 months and once every 4 weeks for 6 months

Tian and Zhong, 2008 [14]	2 weeks	Not reported	Acupuncture (*n* = 116)	Chinese patent medicine (*n* = 112)	61/116 (52.59%)	47/112 (41.96%)	Cure rate, improvement rate

Ling and Chen, 2011 [15]	1 month	Not reported	Acupoint injection (*n* = 30)	Chinese patent medicine (*n* = 30)	18/30 (60%)	15/30 (50%)	Cure rate, improvement rate

Radmayr et al., 2001 [16]	6 months	Not reported	Laser Acupuncture (*n* = 20)	Western medicine: desmopressin (*n* = 20)	13/20 (65%)	15/20 (75%)	Response rate, partial response rate

Radvanska et al., 2011 [17]	5 weeks	Not reported	Laser acupuncture (*n* = 16)	Placebo therapy: without active laser light but with or without skin contact (*n* = 13)	Not reported	not reported	Wet nights/wk, voiding frequency, nocturnal urine production on wet nights MVV (maximal voided volume), AVV (average voided volume)

Yang et al., 2012 [18]	1 month	Not reported	Ear point tapping with medicinal cake-separated moxibustion (*n* = 35)	Chinese patent medicine (*n* = 34)	21/35 (60%)	12/34 (35.3%)	Cure rate, improvement rate

Luo, 2010 [19]	3 months	Not reported	Acupuncture-massage (*n* = 20)	Chinese medicine (*n* = 20)	14/20 (70%)	4/20 (20%)	Cure rate, improvement rate

Tang et al., 2012 [20]	2 weeks	1 month	Massage (*n* = 24)	Chinese medicine (*n* = 24)	16/24 (66.7%)	11/24 (45.8%)	Cure rate, improvement rate

Qiu, 2008 [21]	1 month	Not reported	Ear point tapping (*n* = 31)	Chinese medicine (*n* = 25)	17/31 (54.8%)	15/25 (60%)	Cure rate, improvement rate

Zhu et al., 2003 [22]	3 weeks	3 months	Acupoint injection (*n* = 41)	Western medicine: Meclofenoxate (*n* = 35)	19/41 (46.5%)	6/35 (17.1%)	Cure rate, improvement rate

Zhang, 2010 [23]	1 month	Not reported	Medicinal cake-separated moxibustion with embedded needling (*n* = 40)	Western medicine: desmopressin (*n* = 40)	22/40 (55%)	6/40 (15%)	Cure rate, improvement rate

Chen and Gu, 2003 [24]	2 weeks	Not reported	Acupoint injection (*n* = 40)	Chinese medicine (*n* = 32)	36/40 (90%)	14/32 (43.7%)	Cure rate, improvement rate

Yuksek et al., 2003 [25]	6 months	Not reported	Acupressure (*n* = 12)	Western medicine: oxybutynin (*n* = 12)	10/12 (83.3%)	7/12 (58.3%)	Complete improvement rate, partial improvement rate, follow-up at 15 days and 1, 3, and 6 months

Hong and Zhang, 2009 [26]	1 month	Not reported	Needle warming moxibustion (*n* = 15)	Chinese medicine (*n* = 15)	13/15 (86.7%)	5/15 (33.3%)	Cure rate, improvement rate
